# Unusual presentation of a cutaneous metastasis in the face arising from gastric cancer: a case report

**DOI:** 10.1177/2050313X18795080

**Published:** 2018-09-10

**Authors:** Michael Constantin Kirchberger

**Affiliations:** Department of Dermatology, University Hospital Erlangen, Erlangen, Germany

**Keywords:** Skin metastasis, gastric cancer, Sister Mary Joseph’s nodule

## Abstract

A well-known example of gastrointestinal cancers metastasizing to the skin is Sister Mary Joseph’s nodule, which usually presents as a cutaneous nodule on the umbilicus. In this case, a 91-year-old man was referred to our dermatology clinic for a rapidly growing 3 cm × 2 cm ulcerative nodule at his chin. Biopsy showed skin metastasis originating from a gastric adenocarcinoma. The subcutaneous and cutaneous manifestation of gastric cancer is very rare and associated with a poor prognosis and widespread metastatic disease as presented in this case. However, skin metastasis may be the first clinically apparent sign of underlying systemic malignancy and therefore immediate clarification in case of uncertainty is recommended.

## Introduction

Apart from skin metastases, paraneoplastic phenomena such as acanthosis nigricans and tripe palm are often the herald of an underlying systemic malignancy.^1^ A well-known example of gastrointestinal cancers metastasizing to the skin is Sister Mary Joseph’s nodule (SMJN), which usually presents as cutaneous nodule on the umbilicus. The estimated frequency of SMJN is up to 3% and accounts for 60% of all malignant umbilical tumors.^2^ Gastric cancer more commonly metastasizes to lymph nodes, liver, and peritoneum. Besides SMJN, subcutaneous metastasis from gastric cancer with an incidence rate of 1% is a very rare skin manifestation.^3^ In this presented case, we report a very unusual and rare presentation of cutaneous metastasis in the face arising from gastric cancer.

## Case report

**Figure 1.**
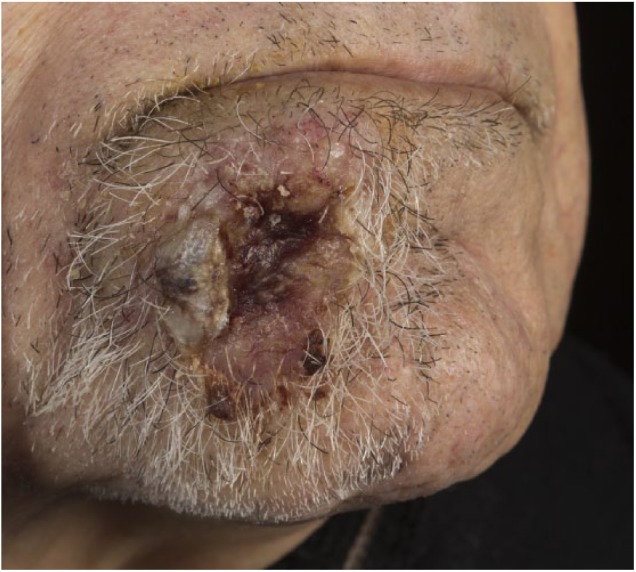
Cutaneous metastasis arising from primary gastric cancer at the chin.

A 91-year-old man was referred to our dermatology clinic for a 3 cm × 2 cm ulcerative nodule at his chin (Figure 1). The lesion had been rapidly growing since approximately 2 months. Biopsy showed skin metastasis originating from a gastric adenocarcinoma. The patient had no fever and did not experience night sweat or weight loss. Computed tomographic scanning of the whole body revealed a gastric tumor with blood vessel infiltration, peritoneal carcinomatosis, pulmonary metastases with pericardium infiltration, and multiple disseminated subcutaneous metastases. The patient refused therapy and died a month after diagnosis.

## Discussion

Subcutaneous and cutaneous manifestation of gastric cancer is very rare and associated with a poor prognosis and widespread metastatic disease as presented in this case. However, skin metastasis may be the first clinically apparent sign for underlying systemic malignancy and therefore immediate clarification in case of uncertainty is recommended. In this patient, early recognition could have been beneficial for treatment course. Nonetheless, surgical resection of skin metastases arising from gastrointestinal cancers is mostly undertaken as palliative treatment to improve the patient’s quality of life and avoid hemorrhage.^3^
